# Prophylactic intervention of probiotics (*L.acidophilus*, *L.rhamnosus* GG) and celecoxib modulate Bax-mediated apoptosis in 1,2-dimethylhydrazine-induced experimental colon carcinogenesis

**DOI:** 10.1186/s12885-018-4999-9

**Published:** 2018-11-13

**Authors:** Leila Kaeid Sharaf, Mridul Sharma, Deepika Chandel, Geeta Shukla

**Affiliations:** Department of Microbiology, Basic Medical Sciences (Block I), South Campus, Panjab University, -160014, Chandigarh, India

**Keywords:** Apoptosis, Colorectal cancer, Gastrointestinal diseases, Celecoxib, Probiotics

## Abstract

**Background:**

Colorectal cancer has been found to be attenuated either with prophylactic manipulation of gut microbiome with probiotics or celecoxib, a non-steroidal anti-inflammatory drug mainly by suppressing early pro-carcinogenic markers in various experimental studies. Therefore, the present study was designed to assess the prophylactic potential of combinatorial administration of probiotics (*Lactobacillus rhamnosus* GG, *Lactobacillus acidophilus*) and celecoxib in experimental colon carcinogenesis.

**Methods:**

Six groups of Spraugue Dawely rats received probiotics *L.rhamnosus* GG or/and *L.acidophilus* in combination with celecoxib one week prior to the inducement of tumor by 1,2-dimethylhydrazine (DMH) and the treatment continued for 18 weeks. Prophylactic potentials of probiotics and celecoxib were determined by employing various methods such as tumor incidence, tumor burden, tumor multiplicity, apoptosis, caspase activity, expression of proto-oncogene K-ras and tumor suppressor p53 gene in colonic tumors.

**Results:**

Interestingly, it was found that one week prior supplementation of both probiotics and celecoxib reduced tumor burden, tumor multiplicity, down-regulated the expression of anti-apoptotic Bcl-2, proto-oncogene K-ras and up-regulated pro-apoptotic Bax as well as tumor suppressor p53 in *L*.*rhamnosus* GG + celecoxib+DMH animals compared with counter controls and DMH-treated.

**Conclusions:**

It can be concluded that such combinatorial approach may be useful in reducing the burden and severity of disease in highly susceptible individuals but needs to be validated clinically.

**Electronic supplementary material:**

The online version of this article (10.1186/s12885-018-4999-9) contains supplementary material, which is available to authorized users.

## Background

Colorectal cancer (CRC), one of the leading causes of cancer related death worldwide is the most common gastrointestinal malignancies and ranks the third most prevalent cancer in male and second in females [1]. CRC is a heterogenous disease occurring either due to genetic mutations, epigenetic changes, chronic inflammation, diet or lifestyle [2]. Generally, in CRC tumorigenesis, the transformation of colorectal epithelium to carcinoma is due to progressive inhibition or evasion of apoptosis, an evolutionally conserved cell suicidal process, to maintain a normal tissue homeostasis in multi cellular organisms [3, 4]. Studies have shown that resistance to apoptosis is a multifactorial process and may be due to either insufficient expression of p53, Bax, Bak or overexpression of anti-apoptotic proteins such as Bcl-2 and Bcl-xL which may be running parallel or functioning independently to Bcl-2 signaling molecules [5].

Adverse effects associated with conventional therapies necessitate the complementary and alternative intervention strategies to reduce the morbidity and enhance the survival rate of cancer patients [6, 7]. More particularly, animal and epidemiological studies as well as clinical trials have shown that the most widely used non-steroidal anti-inflammatory drugs (NSAIDs) prevent intestinal cancer both in mice and human, specifically in high-risk individuals who carry germ-line APC as NSAIDs have the potential to inhibit cyclooxygenase activity, induce apoptosis and crosstalk between intrinsic and extrinsic pathways [8, 9]. Celecoxib, a selective COX-2 inhibitor possesses anti-inflammatory and anti-tumorigenic efficacy and had been clinically approved by FDA for familial adenomatous polyposis (FAP) patients [10, 11]. Although, celecoxib effectively prevents or reduces colon cancer in patients but, due to potential toxic adverse effects its use as a chemopreventive agent is limited [12, 13]. Therefore, natural biointervention agents such as probiotics have been employed as an attractive strategy with prophylactic property and enhanced effectiveness in experimental CRC [13, 14]. Since, colon is the main target of microbial colonization and any perturbation in gut microflora may exert a negative influence on the health or physiology of the host, therefore, probiotics, have been investigated for their ability to beneficially modulate the biomarkers of CRC [14–16]. Moreover, probiotics have been found to enhance the mucosal immune response as well as regulate the apoptosis and cell differentiation in experimental CRC [14, 17, 18]. Though, information pertaining to application of non-steroidal anti-inflammatory drug (Celecoxib) or probiotics alone is available in experimental colon carcinogenesis, yet supplementation of probiotic in conjunction with celecoxib is not available and warrants further investigations. Moreover, in our earlier study, we have found that combination of both probiotics and celecoxib do have anti-neoplastic efficacy in early stage of experimental colon cancer, primarily by inhibiting the initiation and progression of colon cancer mainly due to down-regulation of pro-carcinogenic markers (COX-2, NF-κB, β-catenin) expression [19]. Thus, the present study was under taken to elucidate the molecular modulatory potentials of probiotics in conjunction with celecoxib in experimental colon carcinogenesis employing SD rats with respect to anti-tumorigenesis markers and apoptosis.

## Methods

### Animals

Male Sprague-Dawley (SD) rats (100-200 g) were procured from the inbred population of Central Animal House, Panjab University, Chandigarh, India. Rats were kept in standard polypropylene cages (3 animals per cage) with a wire mesh top and a hygienic bed of husk (regularly changed) in room with 12 h light/dark cycle, constant temperature (24 °C) and humidity, these were acclimatized for one week and provided with normal standard pellet diet and water ad libitum*.* Care and uses of animals were as per the principles and guidelines of the Ethics Committee of the Animal Care of Panjab University, Chandigarh, India, till end of the experimental period (PU/IAEC/S/15/70).

### Study design

#### Induction of colon carcinogenesis

A single dose of DMH (20 mg/kg body weight) was given intraperitoneally (i.p), once in a week to animals and the treatment was continued for 18 weeks [14].

#### Probiotic strains and probiotic dose

For experimental use, 18 h old standard probiotic cultures, *L.rhamnosus* GG MTCC #1408 and *L.acidophilus* NCDC #15 were cold centrifuged at 3500 g for 10 min, washed, and suspended in phosphate buffered saline (PBS, pH 7.2) to contain 1 × 10^9^ lactobacilli/0.1 ml and were fed to animals via oro-gastric gavages [14].

#### Preparation of celecoxib and dose

Celecoxib (6 mg/kg) was prepared in 0.5% carboxymethylcellulose sodium salt (CMC) and was administered via oro-gastric gavage to the animals [11].

### Chemicals

All the chemicals used in the present study were of analytical grade and obtained from standard companies. 1,2-dimethylhydrazine (DMH), dithiothreitol and TRIzol reagent, glutathione peroxidase, cDNA KIT, and primers were procured from Sigma Chemical Company, St. Louis, Minnesota, USA. De Man Rogosa Sharpe (MRS) broth, MRS agar, Hank’s balanced salt solution (HBSS), trypan blue, sodium chloride, potassium chloride, sodium dihydrogen phosphate, potassium dihydrogen phosphate, tri-sodium citrate and tris-hydrochloric acid, ethylenediaminetetraacetic acid EDTA, tris-saturated phenol, agarose, ethidium bromide, acridine orange, DEPC reagent were purchased from Hi-Media Pvt. Ltd. Laboratories Mumbai, India. Caspase 3, 8 and 9 kits were procured from BioVision USA. Celecoxib was procured from Ranbaxy Research Laboratory (Gurgaon, India).

### Experimental procedure

Animals were randomly divided into six groups with six rats each and were given the following treatment in the morning daily at regular intervals in the central animal house.

- **Group I (Control):** Animals were administered with 1 mM EDTA-saline, once a week i.p and 0.5% CMC daily, orally, via orogastric gavage, for 18 weeks.
- **Group II (DMH):** Animals received DMH (20 mg/kg) in 1 mM EDTA-saline i.p, once a week for 18 weeks.
- **Group III (Celecoxib + DMH):** These animals were given celecoxib (6 mg/kg) orally, via gavage, daily for one week. Thereafter, DMH was administered, once a week i.p for 18 weeks along with daily administration of celecoxib.
- **Group IV (*L.acidophilus +*** **Celecoxib + DMH):** Animals received *L.acidophilus* and celecoxib orally, via gavage, daily for one week. Thereafter, a single dose of DMH was given i.p, once a week for 18 weeks along with daily oral feeding of both probiotic and celecoxib.
- **Group V (*L.rhamnosus*** **GG*+*** **Celecoxib + DMH):** Animals were administered with *L.rhamnosus* GG and celecoxib orally daily for one week. After that, a single dose of DMH was given, i.p once a week for 18 weeks along with daily oral administration of probiotic and celecoxib.
- **Group VI (*L.acidophilus + L.rhamnosus*** **GG** ***+*** **Celecoxib + DMH):** Animals were fed orally with *L.acidophilus, L.rhamnosus* GG and celecoxib orally, daily for one week. Thereafter, a single dose of DMH was given i.p once a week for 18 weeks along with daily administration of celecoxib and probiotics.

### Follow up of the animals

After 18 weeks of respective treatment, all animals belonging to various groups were sacrificed by over dose of diethyl ether followed by cervical dislocation to minimize the suffering and colon was removed to assess the tumor range, tumor incidence, tumor burden, tumor multiplicity, apoptosis, caspase activity, expression of proto-oncogene K-ras and tumor suppressor p53 gene in all animals (6/6 per group).

### Monitoring of tumor range, tumor incidence, tumor burden and tumor multiplicity

After cervical dislocation of animals, colon was resected, cleansed and examined macroscopically for tumors. Prophylactic response of probiotics in conjunction with celecoxib was assessed on the basis of tumor range (minimum to maximum number of tumors observed in each group), tumor incidence (percentage of animals having tumors), tumor burden (total number of tumor counted/total number of animals) and tumor multiplicity (total number of tumor counted/ number of tumor bearing animals) [18].

### Apoptotic studies

#### DNA fragmentation

To determine apoptosis, DNA from the colonic tumor of all groups was isolated as per Strauss, 1987 with minor modification. Briefly, 60–70 mg of tumor bearing colonic tissue was minced, suspended in 500 μl digestion buffer (20 mM Tris HCl, 50 mM EDTA and 0.5% Triton) and kept at 50 °C overnight. An equal volume of the Tris-saturated phenol was added to the digested tissue and centrifuged at 8000 g for 15 min. The upper layer formed after centrifugation was subjected to phenol–chloroform–isoamyl alcohol extraction procedure. Finally, DNA was precipitated with chilled ethanol, washed with 70% ethanol, dried and dissolved in Tris–EDTA buffer. Isolated DNA was electrophoresed on 1.2% agarose ethidium bromide gel and analysed by Gel Doc EZ Imager (Bio-Rad, USA).

#### Colonocytes extraction

After sacrificing the animals, entire colon was removed, flushed with Ca^2+^ and Mg^2+^ free-PBS and was cut longitudinally to expose the lumen. The cut colon was placed in warm Ca^2+^ and Mg^2+^ free-Hank’s buffered salt solution (HBSS), 30 mM EDTA, 5 mM dithiothreitol (DTT), 0.1% bovine serum albumin (BSA), incubated at 37 °C on shaker for 15 min and mucosal side was gently scraped. The isolated cells were cold centrifuged at 600 g and washed twice in HBSS containing Ca2+ and Mg^2+^ and 0.1% BSA. The final volume was made up to 2 ml, the cells were counted in a hemocytometer and were adjusted to contain 1 × 10^5^ cells/ml. The cell viability was assessed by trypan blue (0.2%) exclusion method [20].

#### Determination of apoptotic cells by ethidium bromide/Acridine Orange staining

Ethidium bromide/acridine orange (EtBr/AO) staining was used to visualize fluorometric changes, a characteristic of apoptosis in cells. For detection of apoptosis the isolated colonocytes were stained with (EtBr/AO) and observed under a fluorescence microscope [21].

#### Caspase activity

Caspases, − 3, − 8, and − 9 activities were measured in cytosolic fraction of colonic tumors in animals belonging to all groups using commercially available kits (BioVision, Research products, Mountain View, CA). Briefly, cytosol (10 μl containing 50 μg proteins) was mixed in a microtiter ELISA plate with assay buffer and caspase specific substrates DEVD-pNA (para-nitro-aniline) for caspase-3, IETD-pNA for caspase-8 and LEHD-pNA for caspase-9. Samples were incubated at 37 °C for 1–2 h, thereafter absorbance was measured at 405-nm in a microtiter plate reader (Bio-Rad, USA).

### Isolation of RNA

RNA was extracted from colonic tumors, using TRIzol reagent, a mixture of guanidine thiocyanate and phenol in a monophase solution (Sigma Aldrich, USA) and was processed following manufacturer’s protocol. Concentration, yield and purity of the isolated RNA were analyzed by measuring the absorbance at 260/280 nm in spectrophotometer followed by agarose gel electrophoresis.

#### Synthesis of complementary DNA (cDNA)

Complementary DNA (cDNA) was synthesized from RNA isolated from the colonic tumors of animals belonging to all groups using commercially available kit (Sigma Aldrich, USA).

#### Reverse transcriptase polymerase chain reaction (RT-PCR)

RT-PCR is used to detect or quantify the expression of mRNA, from a small concentration of target RNA, PCR was performed using prepared cDNA. The primers sequences for Bax, Bcl-2, β-actin [22], wild type tumor suppressor p53 [23] and oncogene K-ras [24] are given. The primers were synthesized by Sigma Aldrich, USA.

#### Assessment of apoptotic markers (Bcl-2 & Bax)

Reverse transcriptase polymerase chain reaction (RT-PCR) was employed to monitor the expression of (Bcl-2 & Bax) using commercially available primers sequences following manufacturer’s protocol. 2 μg of total RNA was used for RT-PCR reaction from each group. Primers for PCR were designed to amplify the consensus sequence for **Bcl-2** (F) 5′- CCTGTGGATGACTGAGTACC-3′ and (R)5’ GAGACAGCCAGGAGAAATCA 3′, Primer for **Bax** (F) 5’GTTTCATCCAGGATCGAGCAG 3′ and (R) 5’CATCTTCTTCCAGATGGT3’; **p53** (F) 5’GGCTCCTCCCCAACATCTTATC-3’and (R) 5’TCTCCCAGGACAGGCACAAAC3’; **K-ras** (F) 5’ACTTGTGGTAGTTGGCCCT-3′ and (R) 5’TCCCCAGTTCTCATGTACTG3’; **β-actin**(F)5’ATGGAATCCTGTGGCATCCA3’and(R)5’TCCACACAGAGTACTTGCGCTC3’Reverse transcription reaction was set according to manufacturer protocol (Sigma Aldrich, USA).

PCR for **Bcl-2** and **Bax** was performed using following PCR program: 94 °C for 2 min for initial denaturation; then 35 cycles of denaturation at 94 °C for 15 s; annealing at 55 °C for 30 s; extension at 72 °C for 1 min and final elongation at 72 °C for 5 min. The amplified DNA was resolved in 1.8% agarose ethidium bromide gel and analysed by Gel Doc EZ Imager (Bio-Rad, USA).

#### Proto-oncogene K-ras

PCR for **K-ras** gene was performed using following programme: 94 °C for 2 min for initial denaturation; then 35 cycles of denaturation at 94 °C for 15 s; annealing at 57 °C for 30 s; extension at 72 °C for 1 min and final elongation at 72 °C for 5 min. The amplified DNA was resolved in 1.8% agarose ethidium bromide gel and analysed by Gel Doc EZ Imager (Bio-Rad, USA).

#### Tumor suppressor gene p53

PCR for tumor suppressor gene **p53** was performed using following PCR program: 94 °C for 2 min for initial denaturation; then 35 cycles of denaturation at 94 °C for 15 s; annealing at 58 °C for 30 s; extension at 72 °C for 1 min and final elongation at 72 °C for 5 min. The amplified DNA was resolved in 1.8% agarose ethidium bromide gel and analysed by Gel Doc EZ Imager (Bio-Rad, USA).

#### Statistical analysis

Results were expressed as mean ± standard deviation. The inter group variation was assessed by one-way analysis of variance (ANOVA) followed by post Hoc Test Comparison Bonferroni and statistical significance of the results was calculated at *p* < 0.05.

## Results

### Tumor range, tumor incidence, tumor burden and tumor multiplicity

**Fig. 1:**
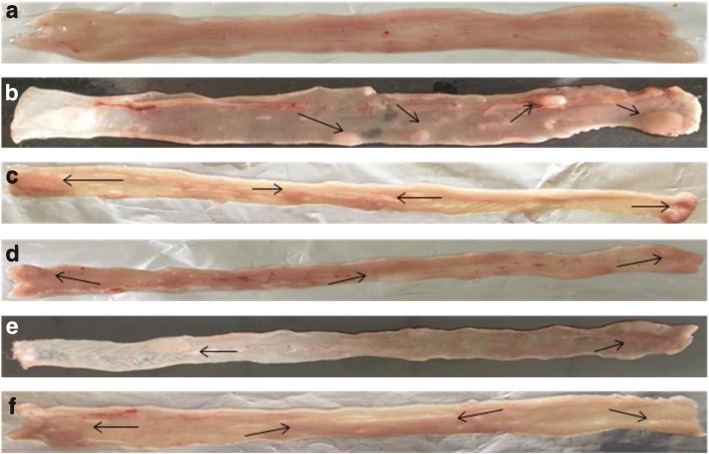
Macroscopic observation of tumors (arrows) in the colon of rats belonging to different groups: **a**) control; **b**) DMH-treated; **c**) celecoxib+DMH; **d**) *L*.*acidophilus* + celecoxib+DMH; **e**) *L*.*rhamnosus* GG + celecoxib+DMH; **f**) *L*.*acidophilus + L*.*rhamnosus* GG + celecoxib+DMH

Macroscopically, it was observed that colon of all animals belonging to DMH-treated groups (IV, V and VI) irrespective of prior treatment of probiotics and celecoxib led to development of sessile polyps that rested on the mucosa (Fig. 1). However, the site and number of tumors were different and was least in *L*.*rhamnosus* GG + celecoxib+DMH (Group V) animals followed by *L.acidophilus* + celecoxib+DMH (IV), *L.acidophilus* + *L.rhamnosus* GG + celecoxib+DMH (VI), celecoxib+DMH (III) respectively compared with DMH-treated (Group II) animals (Fig. 1). Interestingly, it was observed that colon of DMH-treated animals was transparent showing clear prominent, visible nodules compared with opaque colon of other counter control animals (Groups III, IV, V, VI; Fig. 1) (Additional file 1).

More specifically, it was observed that the tumor range belonging to *L*.*rhamnosus*GG + celecoxib+DMH (1–3; Group V) was least followed by *L*.*acidophilus* + *L*.*rhamnosus* GG + celecoxib+DMH (2–4; Group VI), *L*.*acidophilus* + celecoxib+DMH (3–4; Group IV), celecoxib+DMH (3–5; Group III) animals compared with DMH-treated (4–7; Group II) animals (Table 1).

**Table 1:** **Tumor Range** (minimum to maximum number of tumors observed in each group); Tumor Incidence (Percentage of animals having tumors); Tumor Burden (Total number of tumor counted/total number of animals); Tumor Multiplicity (Total number of tumor counted/number of tumor bearing animals) in different groups of animals

Groups of animals	Tumor Range	Tumor Incidence (%)	Tumor Burden	Tumor Multiplicity
DMH	4–7	100	5.8	5.8
celecoxib+DMH	3–5	100	3.9	3.9
*L.acidophilus* + celecoxib +DMH	3–4	100	3.5	3.5
*L.rhamnosus* GG + celecoxib + DMH	1–3	100	2.3	2.3
*L.acidophilus + L.rhamnosus* GG*+* celecoxib *+*DMH	2–4	100	3.2	3.2

Further, it was observed that irrespective of prophylactic treatment of probiotic and celecoxib, animals belonging to all DMH-treated groups (III, IV, V and VI) had 100% incidence of tumor. However, tumor burden and tumor multiplicity was variable among groups and was lowest in *L*.*rhamnosus* GG + celecoxib+DMH (Group V) followed by *L*.*acidophilus* + *L*.*rhamnosus* GG + celecoxib+DMH (Group VI), *L*.*acidophilus* + celecoxib+DMH (Group IV), celecoxib+DMH (Group III) animals compared with DMH-treated (Group II) animals (Table 1).

### Apoptotic study

#### DNA fragmentation

**Fig. 2:**
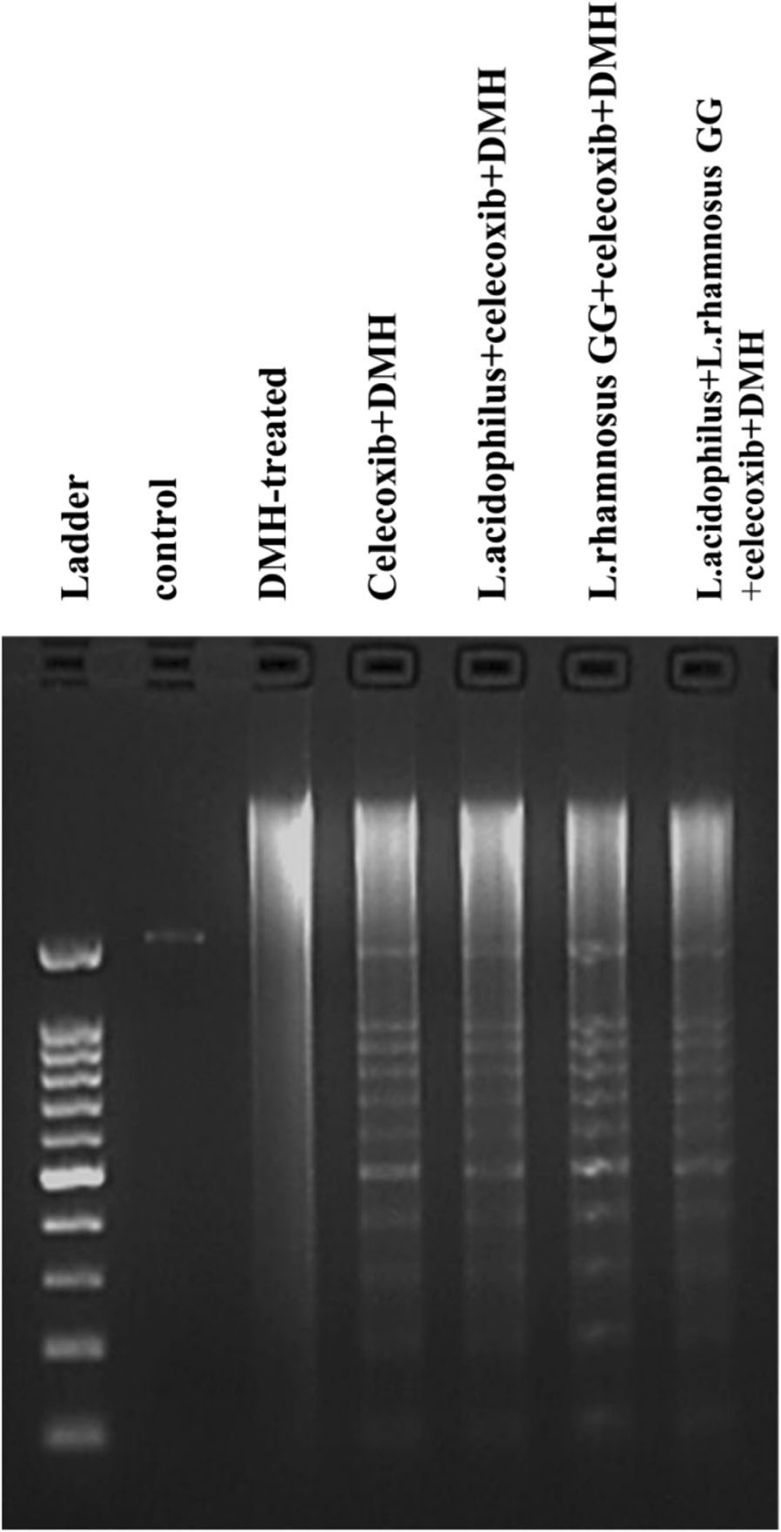
DNA fragmentation in colonic tumors of animals belonging to different groups of animals

DNA fragmentation, a hall mark of apoptosis was performed to assess apoptosis in the colonic tumors of animals belonging to all DMH-treated groups. Interestingly, visible, distinct and complete DNA laddering were observed in animals belonging to celecoxib+DMH, *L.acidophilus* + celecoxib+DMH, *L.rhamnosus* GG + celecoxib+DMH and *L.acidophilus* + *L.rhamnosus* GG + celecoxib+DMH (Fig. 2) whereas only one sharp and intact band in control (Group I) and damaged smeared DNA was observed in DMH-treated (Group II) animals (Fig. 2).

### Quantification of apoptosis

**Fig. 3:**
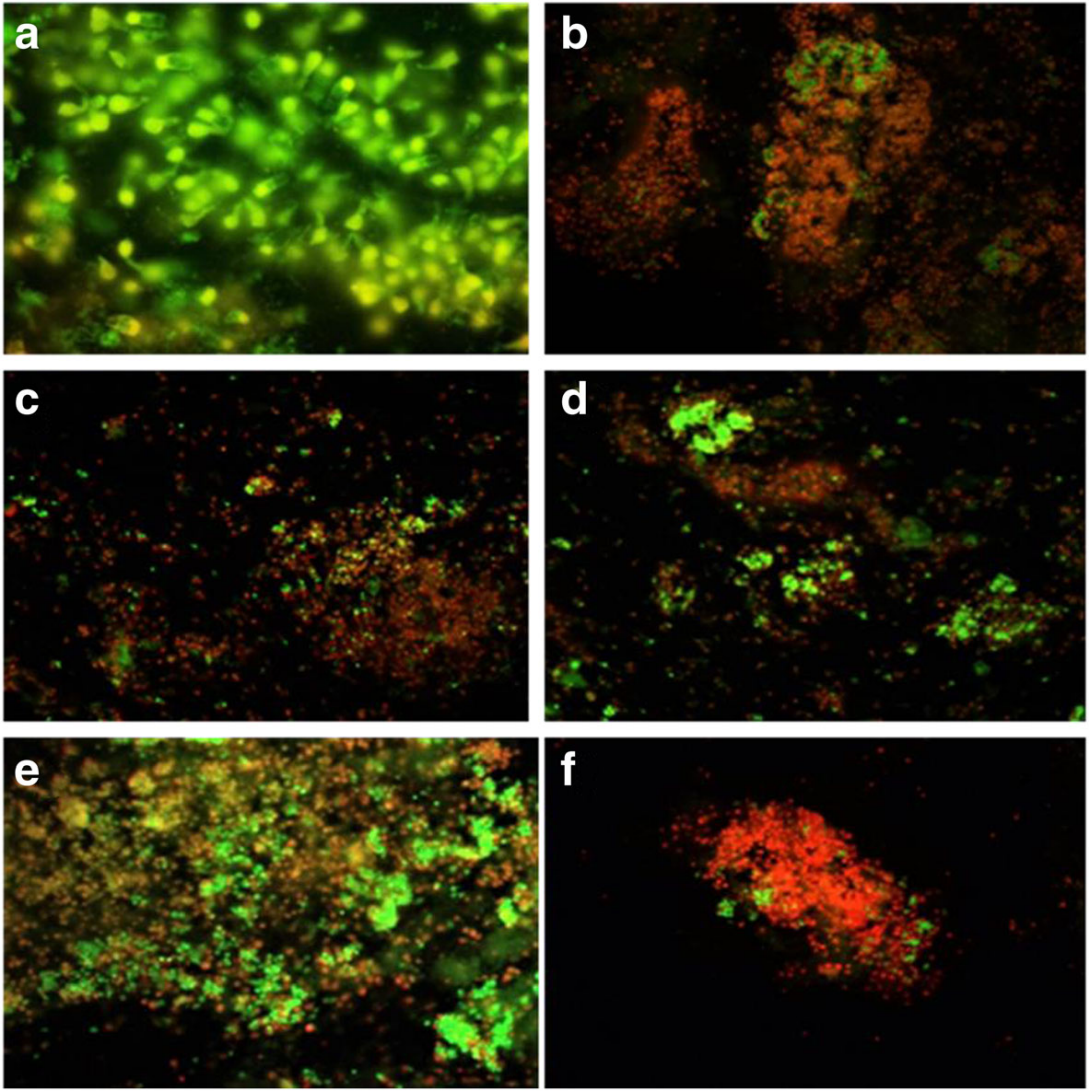
Ethidium bromide/Acridine orange stained colonocytes of animals belonging to different groups: **a**) Control; **b**) DMH-treated; **c**) celecoxib*+*DMH; **d**) *L.acidophilus* + celecoxib+DMH; **e**) *L.rhamnosus* GG + celecoxib+DMH; **f**) *L.acidophilus + L.rhamnosus* GG*+* celecoxib+DMH (400X)

Apoptosis was further quantified by assessing the cell viability of the isolated colonocytes from the colon of animals belonging to all groups by staining with ethidium bromide/acridin orange. It was found that colonocytes isolated from animals belonging to *L*.*rhamnosus* GG + celecoxib+DMH (Group V) had significantly higher (*p* < 0.01) percentage (129%) of apoptotic cells compared with *L*.*acidophilus* + celecoxib+DMH (116%; Group IV), *L*.*acidophilus* + *L*.*rhamnosus* GG + celecoxib+DMH (99.3%; Group VI) and celecoxib+DMH (67%; Group III) animals compared with DMH-treated (Group II) animals (Fig. 3).

### Expression of apoptotic markers, Bcl-2 and Bax

**Fig. 4:**
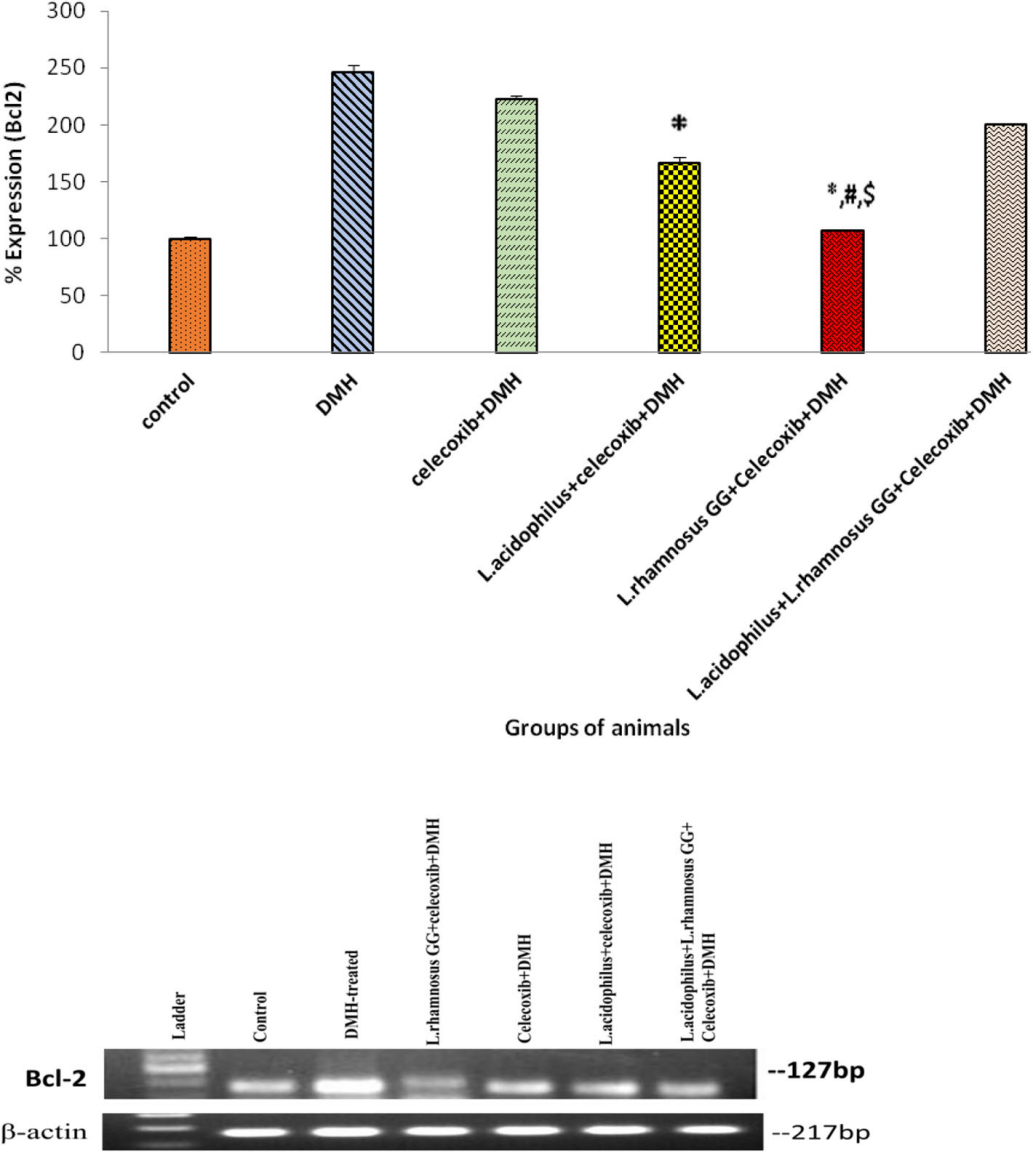
Percentage expression of Bcl-2 in colonic tumors of animals belonging to different groups and their densitometric analysis. Values are expressed as mean ± SD,*p < 0.05 v/s DMH-treated; ^#^p < 0.05 v/s *L.acidophilus* + celecoxib+DMH, ^$^p < 0.05 v/s *L.acidophilus* + *L.rhamnosus* GG + celecoxib+DMH

**Fig. 5:**
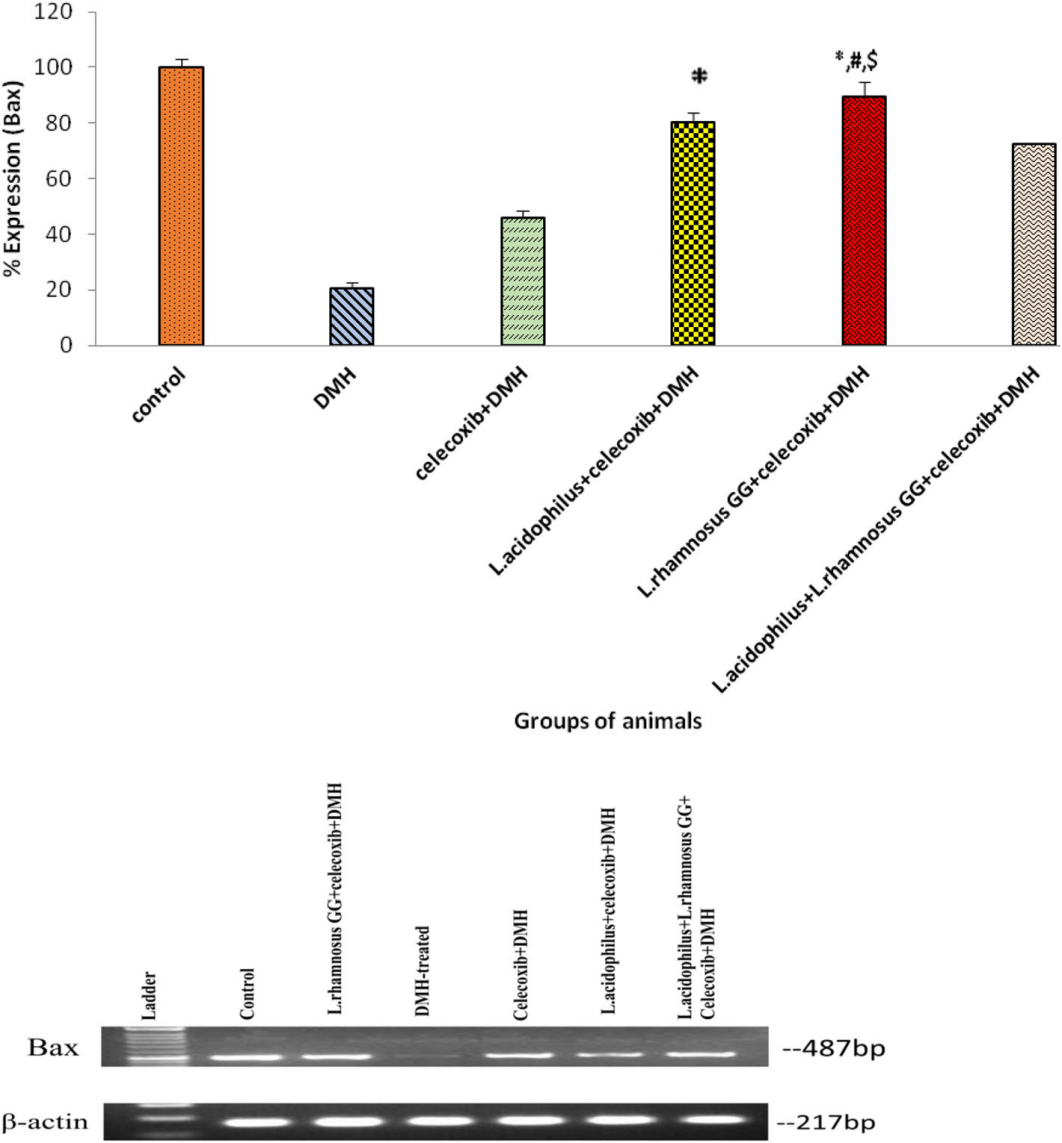
Percentage expression of Bax in colonic tumors of animals belonging to different groups and their densitometric analysis. Values are expressed as mean ± SD, *p < 0.05 v/s DMH-treated; ^#^p < 0.05 v/s *L.acidophilus* + celecoxib+DMH,^$^p < 0.05 v/s *L.acidophilus* + *L.rhamnosus* GG + celecoxib+DMH

It was found that colonic tumors of animals belonging to *L*.*rhamnosus* GG + celecoxib+DMH (Group V) had significantly (*p* < 0.05) down regulation of an anti apoptotic Bcl-2 expression compared with *L.acidophilus* + celecoxib+DMH (Group IV), *L*.*acidophilus* + *L*.*rhamnosus* GG + celecoxib+DMH (Group VI) and DMH-treated (Group II; Fig. 4). More specifically, the pro-apoptotic marker Bax was up-regulated significantly (*p* < 0.05) in *L*.*rhamnosus* GG + celecoxib+DMH (Group V) animals compared with counter control (Groups IV, VI, III) and DMH-treated (Group II) animals (Fig. 5). It was interesting to observe that though, expression of Bax gene was up-regulated significantly (*p* < 0.05) in animals belonging to celecoxib+DMH (Group III) compared with DMH-treated (Group II) animals yet was less than the animals belonging to either *L*.*acidophilus* + celecoxib+DMH (Group IV) or *L*.*acidophilus* + *L*.*rhamnosus* GG + celecoxib+DMH (Group VI; Fig. 5) (Additional file 2).

### Caspase activity

**Fig. 6:**
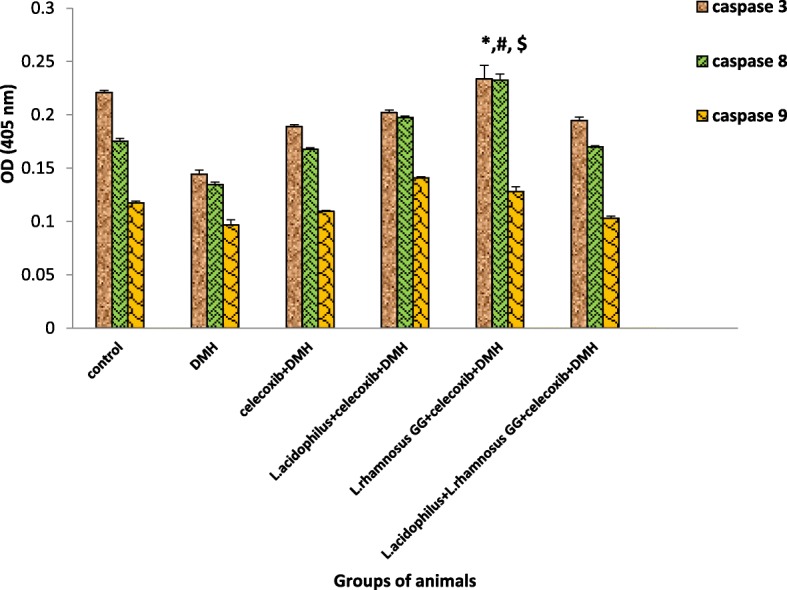
Caspase activity in colonic tumors of animals belonging to different groups. Values are expressed as mean ± SD, *p < 0.05 v/s DMH-treated, ^#^p < 0.05 v/s *L.acidophilus* + celecoxib+DMH, ^$^p < 0.05 v/s *L.acidophilus* + *L.rhamnosus* GG + celecoxib+DMH

It was interesting to observe that caspase-9 activity was significantly higher (*p* < 0.05) in animals belonging to *L.acidophilus* + celecoxib+DMH (IV) followed by *L.rhamnosus* GG + celecoxib+DMH-treated (Group V) compared to DMH-treated (Group II) animals. However, activity of caspase-8 increased significantly (p < 0.05) in *L.rhamnosus* GG + celecoxib+DMH (Group V) animals followed by *L*.*acidophilus* + celecoxib+DMH (Group IV), *L*.*acidophilus* + *L*.*rhamnosus* GG + celecoxib+DMH (Group VI) and celecoxib+DMH (Group III) respectively compared with DMH-treated (Group II) animals (Figure 6). Further, it was observed that, *L.rhamnosus* GG + celecoxib+DMH-treated (Group V) animals had significantly (*p* < 0.05) enhanced level of caspase-3 activity compared to DMH-treated animals (Group II) while, no significant (*p* < 0.05) difference was observed in the activity of caspase-3 in animals belonging to *L.acidophilus* + celecoxib+DMH-treated (IV), *L.acidophilus* + *L.rhamnosus* GG + celecoxib+DMH-treated (Group VI) and celecoxib+DMH-treated (Group III) but was more than DMH-treated (Group II) animals (Fig. 6).

### Expression of proto-oncogene K-ras and tumor suppressor gene p53

**Fig. 7:**
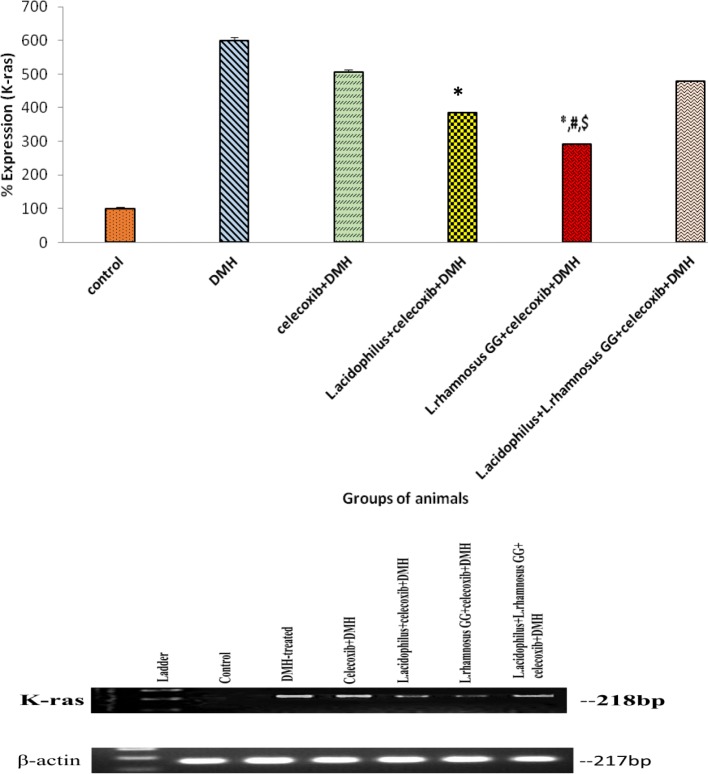
Percentage expression of K-ras in colonic tumors of animals belonging to different groups and their densitometric analysis. Values are expressed as mean ± Standard deviation, *p < 0.01 v/s DMH-treated; ^#^*p* < 0.01 v/s *L.acidophilus* + celecoxib+DMH, ^$^*p* < 0.01 v/s *L.acidophilus* + *L.rhamnosus* GG + celecoxib+DMH

**Fig. 8:**
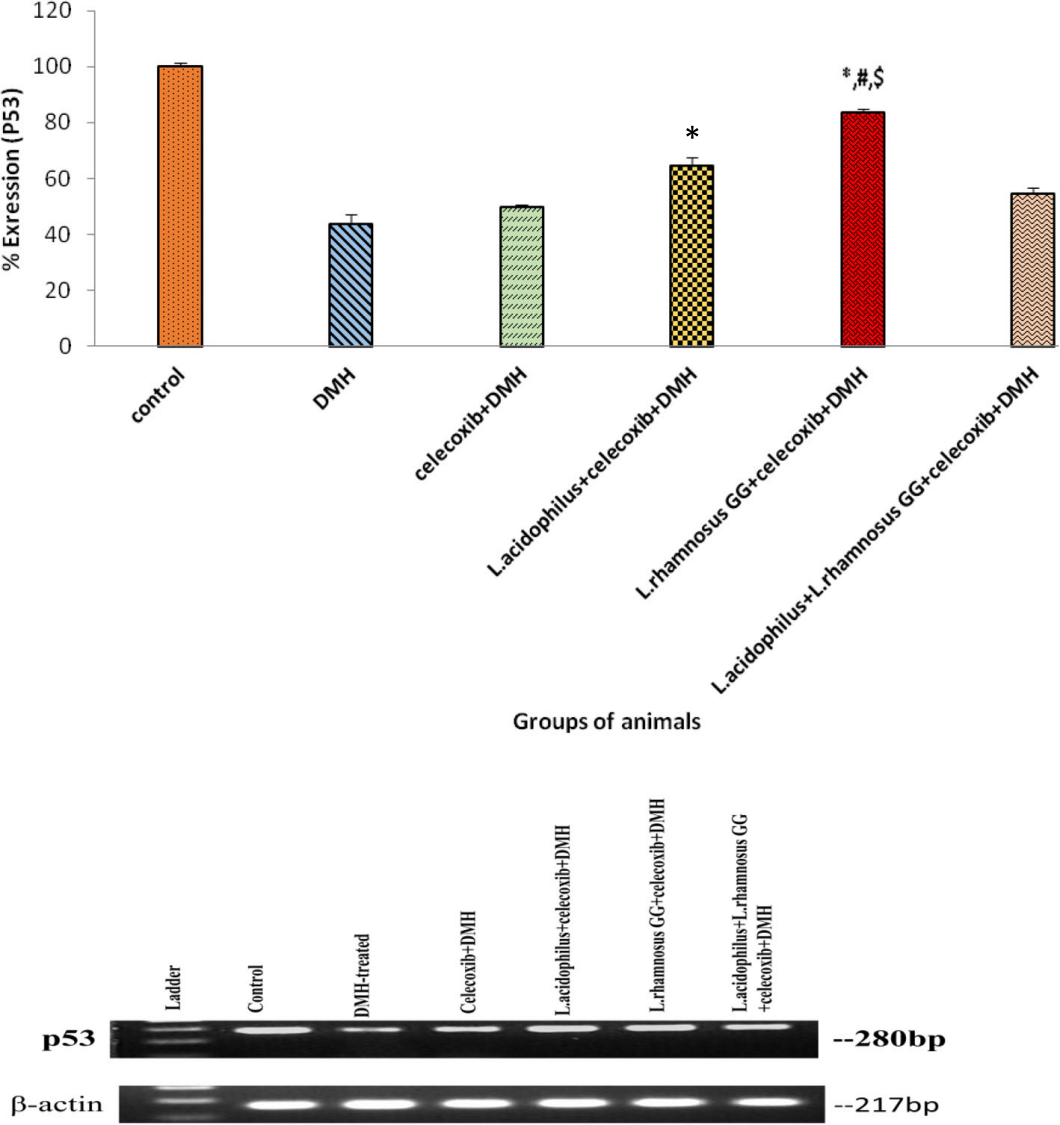
Percentage expression of p53 in colonic tumors of animals belonging to different groups and their densitometric analysis. Values are expressed as mean ± SD, *p < 0.05 v/s DMH-treated; ^#^*p* < 0.05 v/s *L.acidophilus* + celecoxib+DMH, ^$^*p* < 0.05 v/s *L.acidophilus* + *L.rhamnosus* GG + celecoxib+DMH

Densitometric analysis of colonic tumors of animals belonging to all groups showed that prior supplementation of both probiotics and celecoxib down-regulated the expression of K-ras significantly (*p* < 0.01) up-regulated the expression of wild type p53 in animals belonging to all treated groups but maximum effect was observed in *L*.*rhamnosus* GG + celecoxib+DMH (Group V) compared with counter controls and DMH-treated animals (Figs. 7 and 8). However, no significant difference in the expression K-ras and p53 was observed in animals belonging to celecoxib+DMH (Group III) and *L*.*acidophilus* + *L*.*rhamnosus* GG + celecoxib+DMH (Group VI; Figs. 7 and 8).

## Discussions

Apoptosis, a programmed cell death is involved in the maintenance of homeostasis of the body and impaired regulation of apoptosis may play important role in the etiology of many diseases due to which it is emerging as an important mechanism in eliminating the damaged cells by implementation of various prophylactic agents [25]. Moreover, earlier studies have shown that prior supplementation of either probiotics or celecoxib alone modulated DMH-induced experimental CRC [14, 16, 26]. Additionally, we have found that prior administration of both probiotics and celecoxib before the induction of CRC with DMH for six weeks reduced the expression of COX-2, NF-κB and β-catenin, the pro-cacinogenic markers suggesting that tumor initiation steps can be modulated [19]. Therefore, to understand the molecular basis of the combinatorial administration of probiotics and celecoxib in experimental CRC the present study was designed to assess the effect of tumor modulation with respect to apoptosis vis-à-vis expression of p53 and K-ras gene.

The present 18 weeks in vivo experimental colon cancer model demonstrated that in spite of regular administration of both probiotics and celecoxib, DMH treatment led to development of tumor in all animals but the tumor burden and multiplicity were reduced in animals belonging to *L.rhamnosus* GG + celecoxib+DMH. This may be due to the ability of probiotic *L.rhamnosus* GG and celecoxib to inhibit or reduce tumors and coroborates with earlier studies [16, 26]. More specifically, we have observed that combination of probiotic *L.rhamnosus* GG and celecoxib had better anti tumorigenic potential than when given individually [19]. As celecoxib reduced the inflammatory markers, and probiotic *L.rhamnosus* GG modified the gut micro-environment and microbiome. Further, probiotics attenuated the production of procarcinogenic enzymes by altering the microbiome and enhanced the production of various antimicrobial substances, such as bacteriocins, organic acids, reuterin, hydrogen peroxide and de-conjugated bile acids [14, 27, 28]. Additionally, the observed reduced tumors may be due to better mucosal immune response as probiotics do activate various immune cells that lead to production of cytokines having anti-inflammatory and antitumorigenic potential [16]. It has also been demonstrated that probiotic *L.rhamnosus* GG induced the macrophage activation, nitric oxide production by macrophages and significantly increased the production of TNF-α which can be cytotoxic or cytostatic to tumor cells [29, 30].

It was also observed that prior supplementation of probiotics along with celecoxib to DMH-treated animals led to increased apoptosis suggesting their ability to induce apoptosis in colonic tumors due to increased Bax expression that directly binds to or neutralize Bcl-2 vis-à-vis activating caspases. However, earlier studies have also shown the effect of probiotics and celecoxib alone on the induction of apoptosis in experimental colon carcinogenesis and reduced expression of Bcl-2 and increased Bax [16, 26, 31].

Most notably, in present study, it was found that the combinatorial supplementation of probiotics and celecoxib to animals one week prior to induction of CRC down-regulated the expression of proto-oncogene K-ras and up-regulated tumor suppressor p53 and corroborate with earlier observations [16, 32]. This may be due to combined effect of both probiotics and celecoxib that might have reduced the cell proliferation by activation of tumor suppressor gene and maintaining the cell activity and cell cycle. Further, probiotic may have modulated the mucosal immunity by interacting with M cells and Toll-like receptor-2 mediated transcytosis [33, 34]. It also appears that constellation of signals is involved in the protection offered by both probiotics and celecoxib in ameliorating the CRC but role of other biomolecules could not be ruled out.

**Fig. 9:**
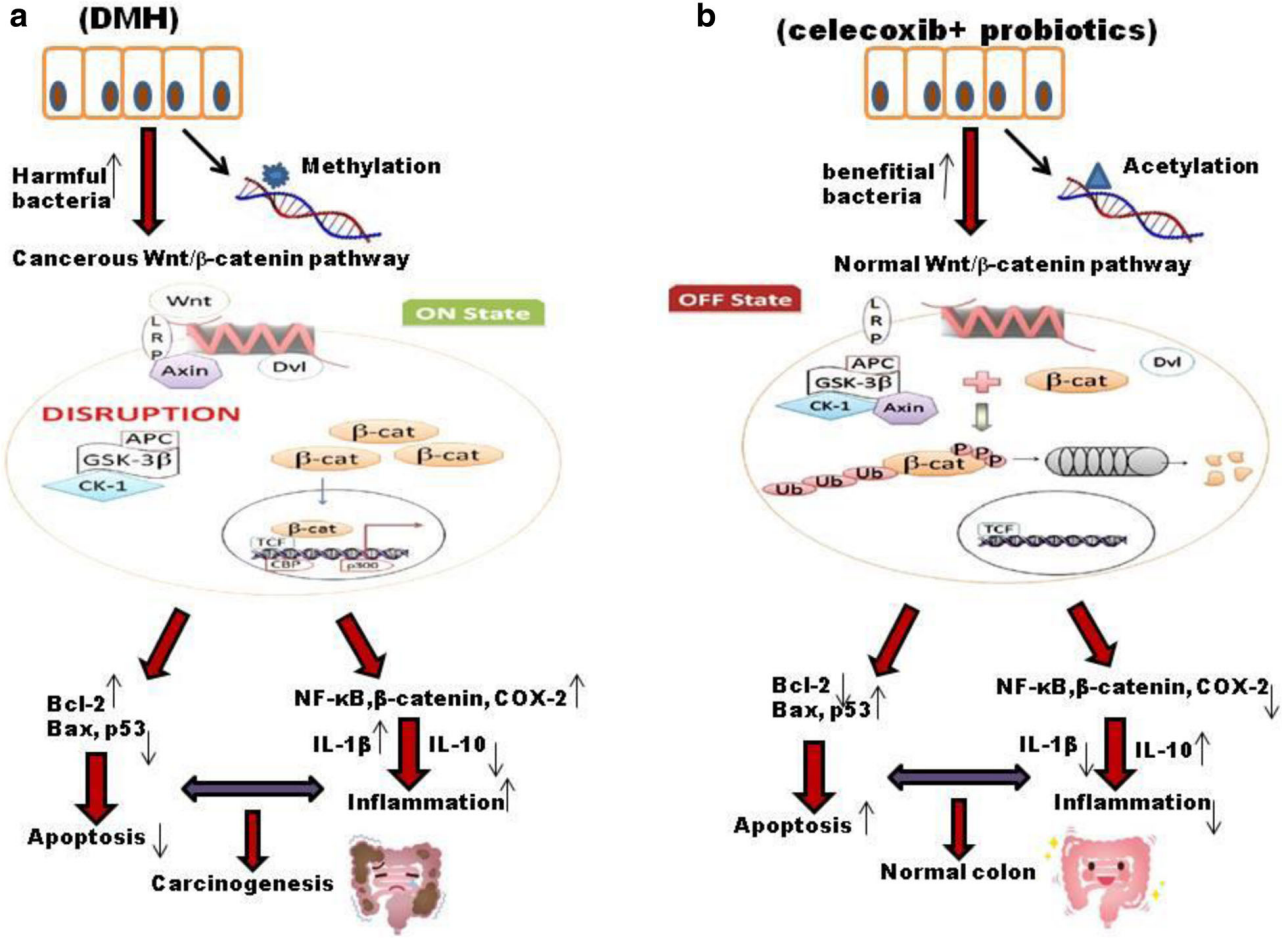
Diagrammatic representation of occurrence of colorectal cancer (**a**) and various modulatory mechanisms of combinatorial administration of probiotics and celecoxib (**b**) in CRC with respect to different biomarkers. (**a**) DMH-induced DNA methylation activates Wnt pathway which is accelerated by over expression of pro-inflammatory markers. Further, presence of harmful bacteria and production of toxic metabolites along with reduced pro-apoptotic markers leads to carcinogenesis. (**b**) Administration of probiotics and celecoxib modulate Wnt signaling pathway and ameliorate gut microbiome which could lead to reduced inflammation and enhanced apoptotic markers subsequently preventing carcinogenesis

Based on the present and earlier observations antitumorigenic and modulatory effect of probiotics in conjunction with celecoxib to colonic tumors against DMH-induced colon carcinogenesis is due to dynamic interplay of several regulatory molecules and modified gut microenvironment. Such combinatorial intervention may have helped in the maintenance of intestinal integrity and enhanced immune-response leading to reduced DNA damage. The anti-inflammatory potential of celecoxib resulted into reduced colonic tumors due to down-regulation of K-ras and up-regulation of p53 which can directly induce Bax mediated apoptosis (Fig. 9).

## Conclusion

It was observed that prior administration of probiotic and celecoxib in experimental colon carcinogenesis attenuated DMH-induced colonic tumors by modifying gut microenvironment and up-regulating tumor suppressor p53 and pro-apoptotic Bax mediated apoptosis. Thus, suggesting the promising prophylactic biointervention for CRC, particularly in highly susceptible individuals but, a significant amount of further clinical research needs to be carried out.

## Additional Files

Additional file 1: Raw Data for table: Calculations for tumor incidence, burden and multiplicity (PDF 293 kb)

Additional file 2: Excel Data for figures: Excel sheets showing data for expression of K-ras, p53, Bax, Bcl-2 and caspase-3, 8, 9 (XLSX 11 kb)

## Abbreviations

APC: Adenomatous polyposis coli
AVE OD: Average of Optical Density
AVE: Average
BSA: Bovine serum albumin
FDA: Food and Drug Administration
NaOH: Sodium hydroxide
St D: Standard Deviation
