# Supplementary material for: Prophylactic intervention of probiotics (L.acidophilus, L.rhamnosus GG) and celecoxib modulate Bax-mediated apoptosis in 1,2-dimethylhydrazine-induced experimental colon carcinogenesis
Source: BMC Cancer. 2018 Nov 13;18:1111. doi: 10.1186/s12885-018-4999-9 (PMC6234654; doi:10.1186/s12885-018-4999-9)
Supplement: Supplementary file 1 — Raw Data for table: Calculations for tumor incidence, burden and multiplicity (PDF 293 kb) [file 12885_2018_4999_MOESM1_ESM.pdf]

Parameters for Long term study:

(E) 1. Estimation of tumor incidence, burden, and multiplicity.

→ cervical Dislocation

↓

colon resected and cleansed and examined for tumor

⇒ Tumor incidence = percentage of animals with tumors

⇒ Tumor burden = $\frac{\text{number of tumors counted}}{\text{total number of animals}}$

⇒ Tumor multiplicity = $\frac{\text{Total no. of tumors counted}}{\text{Tumor bearing animals.}}$

Tumor incidence : 100%

Tumor burden :

Group		Tumor burden		Tumor multiplicity
① control : 0		Tumor burden : 0		Tumor multiplicity : 0
② LA+DMH+Drug:	~	$\frac{21}{6} = 3.5$	~	3.5
③ LGG+DMH+Drug :	~	$\frac{14}{6} = 2.3$	~	2.3
④ LA+LGG+DMH+Drug :	~	$\frac{19}{6} = 3.16$	~	3.16
⑤ LA+GG+Drug	~	0		0
⑥ DMH	~	$\frac{35}{6} = 5.8$	~	5.8
